# Supplementary material for: Mito-SiPE is a sequence-independent and PCR-free mtDNA enrichment method for accurate ultra-deep mitochondrial sequencing
Source: Commun Biol. 2022 Nov 19;5:1269. doi: 10.1038/s42003-022-04182-2 (PMC9675811; doi:10.1038/s42003-022-04182-2)
Supplement: Supplementary file 2 — Supplementary Information [file 42003_2022_4182_MOESM2_ESM.pdf]

S1

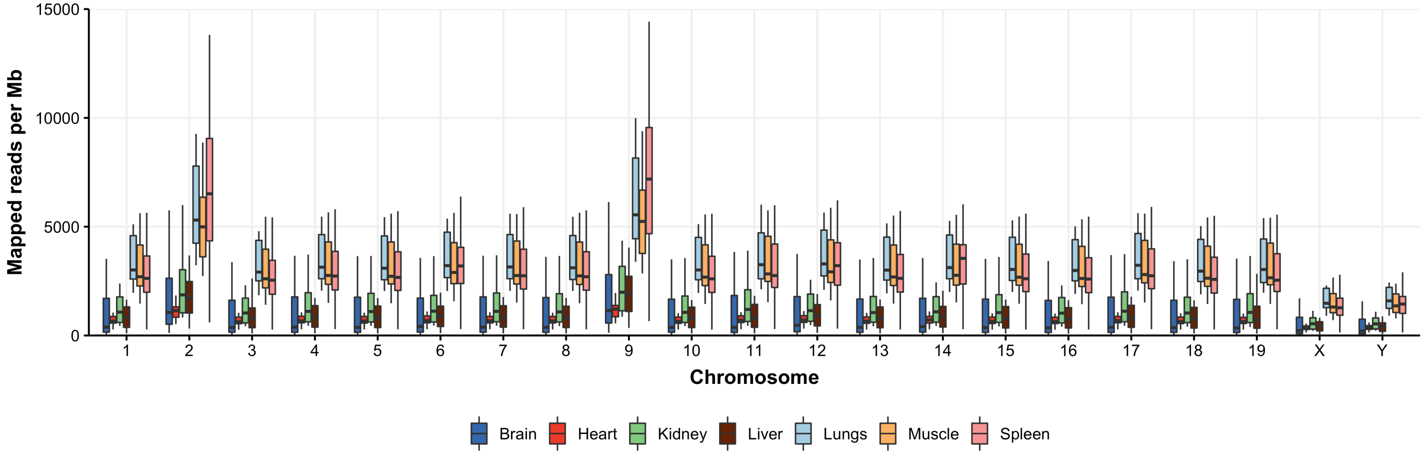

Supplementary figure 1. The distribution of nuclear contamination across the genome shown as boxplots for each tissue. The upper and lower hinges of the boxplot represent the 75th and 25th percentiles, respectively. The middle hinge represents the median.

S2

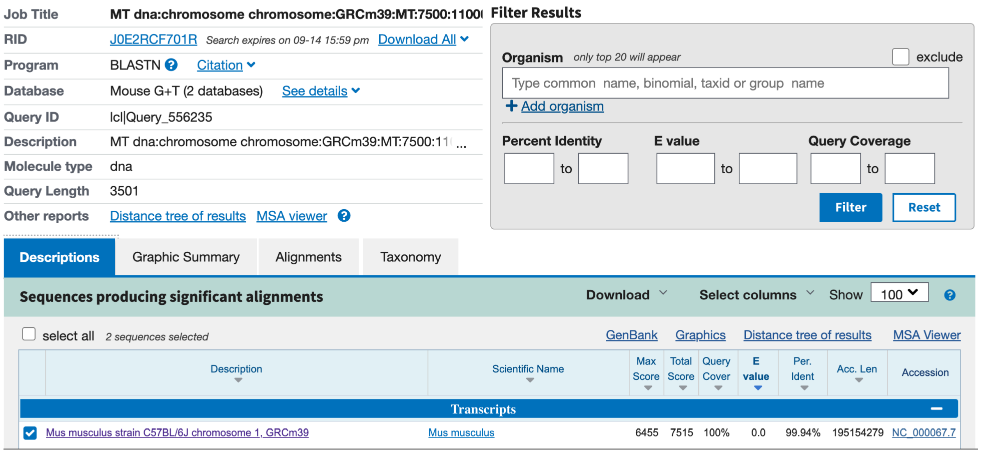

Supplementary figure 2. Sequence alignment results between NUMT on chromosome 1 and its homologous region on chromosome 1 which shows 99.94% sequence identity.

S3

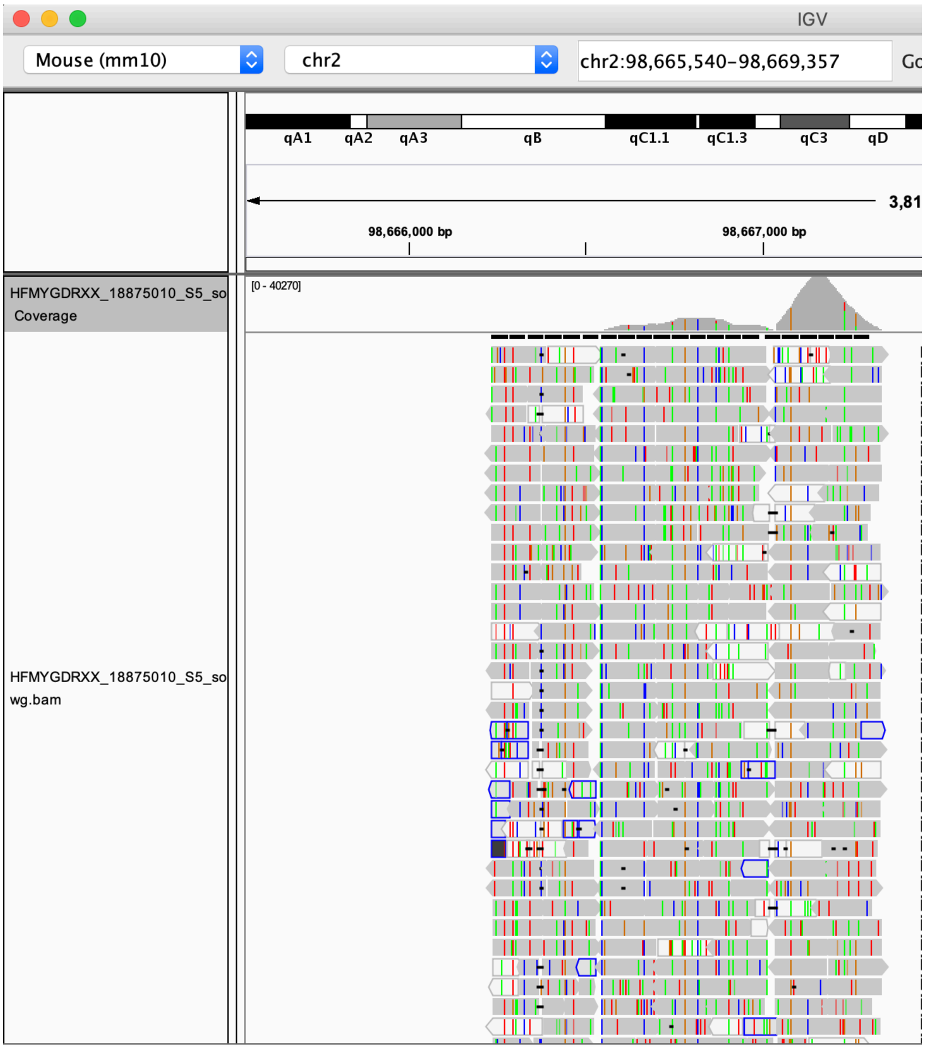

Supplementary figure 3. Integrated genome viewer (IGV) image of high-coverage region on chromosome 2. This image shows the region that reads have aligned to on chromosome 2 due to its sequence similarity with other regions in the genome.

S4

**Program**	BLASTN Citation
**Database**	refseq_genomes (GPIPE/10090/109/ref_top_level) See details
**Query ID**	lcl|Query_57980
**Description**	2 dna:chromosome chromosome:GRCm39:2:98666230:9 ...
**Molecule type**	dna
**Query Length**	1133
**Other reports**	Distance tree of results MSA viewer

Type common name, binomial, taxid or group name

+ Add organism

Percent Identity ___ to ___ | E value ___ to ___ | Query Coverage ___ to ___

Filter | Reset

Descriptions | Graphic Summary | Alignments | Taxonomy

Sequences producing significant alignments Download | Select columns | Show 100

select all *20 sequences selected* GenBank | Graphics | Distance tree of results | MSA Viewer

Description	Scientific Name	Max Score	Total Score	Query Cover	E value	Per. Ident	Acc. Len	Accession
Mus musculus strain C57BL/6J chromosome 2, GRCm39	Mus musculus	2093	2747	100%	0.0	100.00%	181755017	NC_000068.8
Mus musculus strain C57BL/6J chromosome 10, GRCm39	Mus musculus	268	3010	94%	3e-69	76.31%	130530862	NC_000076.7
Mus musculus strain C57BL/6J chromosome 18, GRCm39	Mus musculus	230	1864	93%	2e-57	75.45%	90720763	NC_000084.7
Mus musculus strain C57BL/6J chromosome 14, GRCm39	Mus musculus	219	2722	89%	4e-54	75.39%	125139656	NC_000080.7
Mus musculus strain C57BL/6J chromosome 17, GRCm39	Mus musculus	217	544	43%	1e-53	75.41%	95294699	NC_000083.7
Mus musculus strain C57BL/6J chromosome 5, GRCm39	Mus musculus	211	1468	87%	6e-52	75.39%	151758149	NC_000071.7
Mus musculus strain C57BL/6J chromosome 1, GRCm39	Mus musculus	209	3276	90%	2e-51	75.20%	195154279	NC_000067.7
Mus musculus strain C57BL/6J chromosome 11, GRCm39	Mus musculus	204	2222	88%	1e-49	74.95%	121973369	NC_000077.7
Mus musculus strain C57BL/6J chromosome 3, GRCm39	Mus musculus	202	2785	80%	4e-49	78.01%	159745316	NC_000069.7
Mus musculus strain C57BL/6J chromosome 13, GRCm39	Mus musculus	198	3253	89%	5e-48	76.37%	120883175	NC_000079.7
Mus musculus strain C57BL/6J chromosome 16, GRCm39	Mus musculus	198	1414	91%	5e-48	74.20%	98008968	NC_000082.7
Mus musculus strain C57BL/6J chromosome 12, GRCm39	Mus musculus	196	1437	87%	2e-47	76.80%	120092757	NC_000078.7
Mus musculus strain C57BL/6J chromosome X, GRCm39	Mus musculus	195	4409	89%	6e-47	76.09%	169476592	NC_000086.8
Mus musculus strain C57BL/6J chromosome 7, GRCm39	Mus musculus	193	2605	89%	2e-46	76.41%	144995196	NC_000073.7
Mus musculus strain C57BL/6J chromosome 4, GRCm39	Mus musculus	191	2244	89%	8e-46	74.30%	156860686	NC_000070.7
Mus musculus strain C57BL/6J chromosome 6, GRCm39	Mus musculus	191	2005	73%	8e-46	76.47%	149588044	NC_000072.7
Mus musculus strain C57BL/6J chromosome 8, GRCm39	Mus musculus	187	1137	79%	1e-44	74.00%	130127694	NC_000074.7
Mus musculus strain C57BL/6J chromosome 9, GRCm39	Mus musculus	187	1287	79%	1e-44	74.70%	124359700	NC_000075.7
Mus musculus strain C57BL/6J chromosome 15, GRCm39	Mus musculus	176	1493	87%	2e-41	73.53%	104073951	NC_000081.7
Mus musculus strain C57BL/6J chromosome 19, GRCm39	Mus musculus	176	332	44%	2e-41	74.13%	61420004	NC_000085.7

Supplementary figure 4. BLAST results table that shows the high level of sequence identity between the repetitive region on chromosome 2 and similar regions on other chromosomes in the mouse genome.

S5

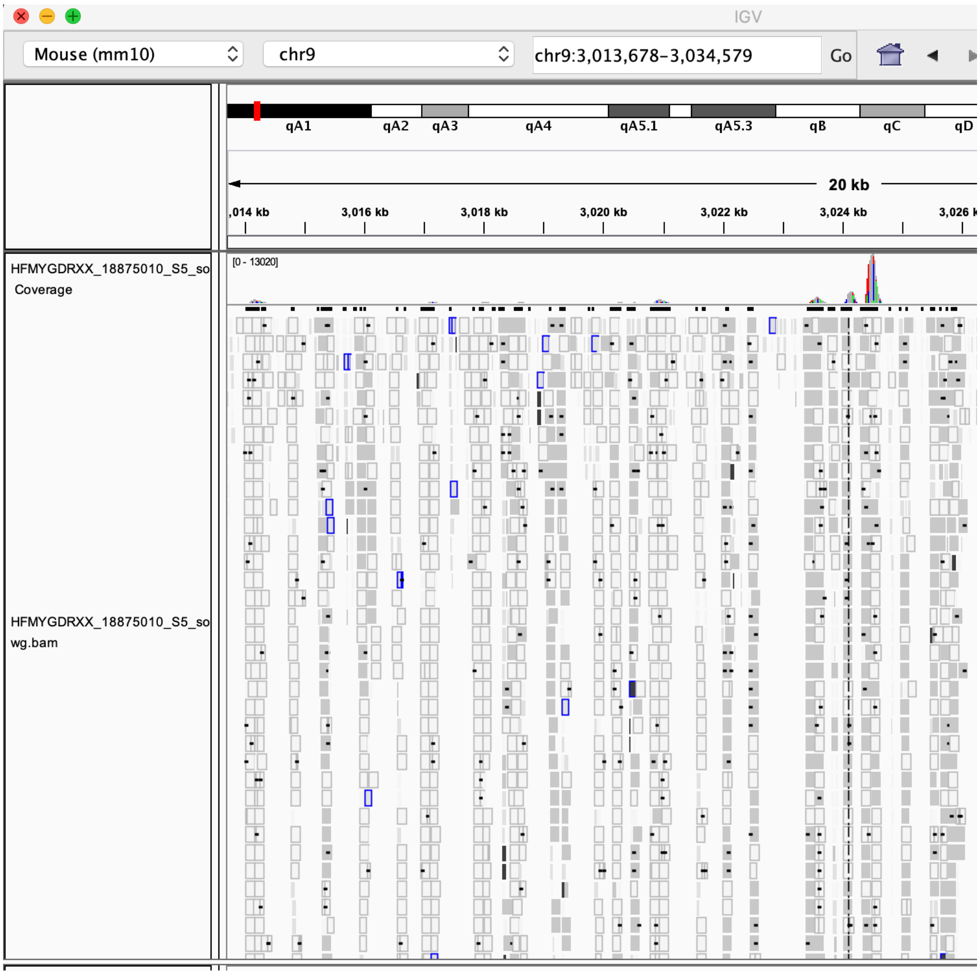

Supplementary figure 5. Integrated genome viewer (IGV) image of high-coverage region on chromosome 9. This image shows the region that reads have aligned to on chromosome 9 due to its sequence similarity with other regions in the genome.

S6

Results for	2:lcl|Query_57981 9 dna:chromosome chromosome:GRCm39:9:3034
Program	BLASTN Citation
Database	refseq_genomes (GPIPE/10090/109/ref_top_level) See details
Query ID	lcl|Query_57981
Description	9 dna:chromosome chromosome:GRCm39:9:3034866:30 ...
Molecule type	dna
Query Length	395
Other reports	Distance tree of results MSA viewer

Organism *only top 20 will appear* exclude

Type common name, binomial, taxid or group name

+ Add organism

Percent Identity to E value to Query Coverage to

Filter Reset

Descriptions Graphic Summary Alignments Taxonomy

Sequences producing significant alignments Download Select columns Show 100

select all *18 sequences selected* GenBank Graphics Distance tree of results MSA Viewer

Description	Scientific Name	Max Score	Total Score	Query Cover	E value	Per. Ident	Acc. Len	Accession
Mus musculus strain C57BL/6J chromosome 9, GRCm39	Mus musculus	730	1.299e+05	100%	0.0	100.00%	124359700	NC_000075.7
Mus musculus strain C57BL/6J unplaced genomic scaffold, GRCm39 MSCHRUN_CTG23	Mus musculus	641	1.435e+05	100%	0.0	95.95%	114452	NT_187064.1
Mus musculus strain C57BL/6J chromosome 2, GRCm39	Mus musculus	630	4122	100%	1e-178	95.44%	181755017	NC_000068.8
Mus musculus strain C57BL/6J chromosome 14, GRCm39	Mus musculus	606	11865	100%	2e-171	94.43%	125139656	NC_000080.7
Mus musculus strain C57BL/6J unplaced genomic scaffold, GRCm39 MSCHRUN_CTG10	Mus musculus	580	7602	100%	1e-163	93.16%	23629	NT_166476.1
Mus musculus strain C57BL/6J unplaced genomic scaffold, GRCm39 MSCHRUN_CTG18	Mus musculus	529	5889	100%	5e-148	91.05%	28772	NT_166473.1
Mus musculus strain C57BL/6J chromosome 12, GRCm39	Mus musculus	424	3006	100%	2e-116	97.20%	120092757	NC_000078.7
Mus musculus strain C57BL/6J unplaced genomic scaffold, GRCm39 MSCHRUN_CTG6	Mus musculus	331	7479	100%	1e-88	82.43%	38659	NT_166467.1
Mus musculus strain C57BL/6J chromosome X unlocalized genomic scaffold, GRCm39 MMCHRX_RAN...	Mus musculus	300	5597	100%	4e-79	80.85%	559103	NT_165789.3
Mus musculus strain C57BL/6J chromosome 3, GRCm39	Mus musculus	279	3106	100%	5e-73	79.95%	159745316	NC_000069.7
Mus musculus strain C57BL/6J unplaced genomic scaffold, GRCm39 MSCHRUN_CTG9	Mus musculus	270	1699	99%	3e-70	79.44%	24668	NT_166474.1
Mus musculus strain C57BL/6J chromosome 4, GRCm39	Mus musculus	265	1285	100%	1e-68	79.35%	156860686	NC_000070.7
Mus musculus strain C57BL/6J unplaced genomic scaffold, GRCm39 MSCHRUN_CTG15	Mus musculus	250	2009	100%	4e-64	79.09%	21240	NT_166480.1
Mus musculus strain C57BL/6J chromosome 13, GRCm39	Mus musculus	241	457	73%	2e-61	95.36%	120883175	NC_000079.7
Mus musculus strain C57BL/6J chromosome 8, GRCm39	Mus musculus	207	207	73%	3e-51	79.93%	130127694	NC_000074.7
Mus musculus strain C57BL/6J chromosome 16, GRCm39	Mus musculus	180	576	73%	5e-43	82.71%	98008968	NC_000082.7
Mus musculus strain C57BL/6J chromosome 11, GRCm39	Mus musculus	172	330	99%	9e-41	75.95%	121973369	NC_000077.7
Mus musculus strain C57BL/6J chromosome 18, GRCm39	Mus musculus	161	161	44%	2e-37	84.48%	90720763	NC_000084.7

Supplementary figure 6. BLAST results table that shows the high level of sequence identity between the repetitive region on chromosome 9 and similar regions on other chromosomes in the mouse genome.

S7

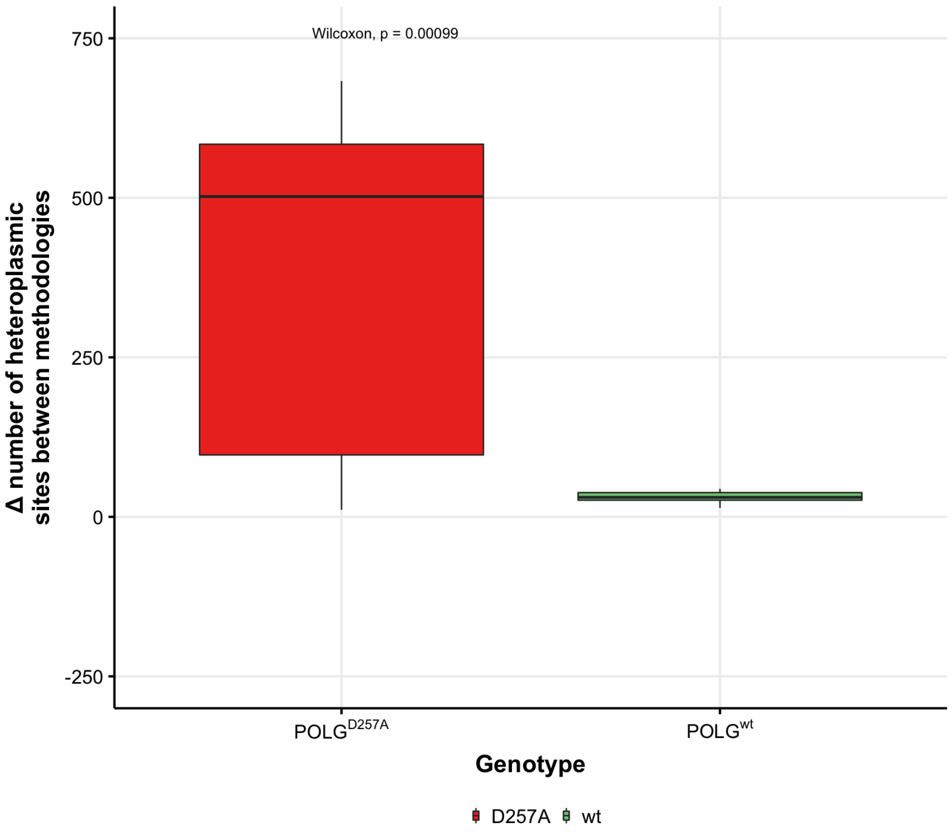

Supplementary figure 7. The difference in number of heteroplasmic sites found between lrPCR and mtDNA preparations in *Polg*$^{D257A/D257A}$ and *Polg*$^{wt/wt}$ tissues shown as boxplots. The upper and lower hinges of the boxplot represent the 75th and 25th percentiles, respectively. The middle hinge represents the median.

S8

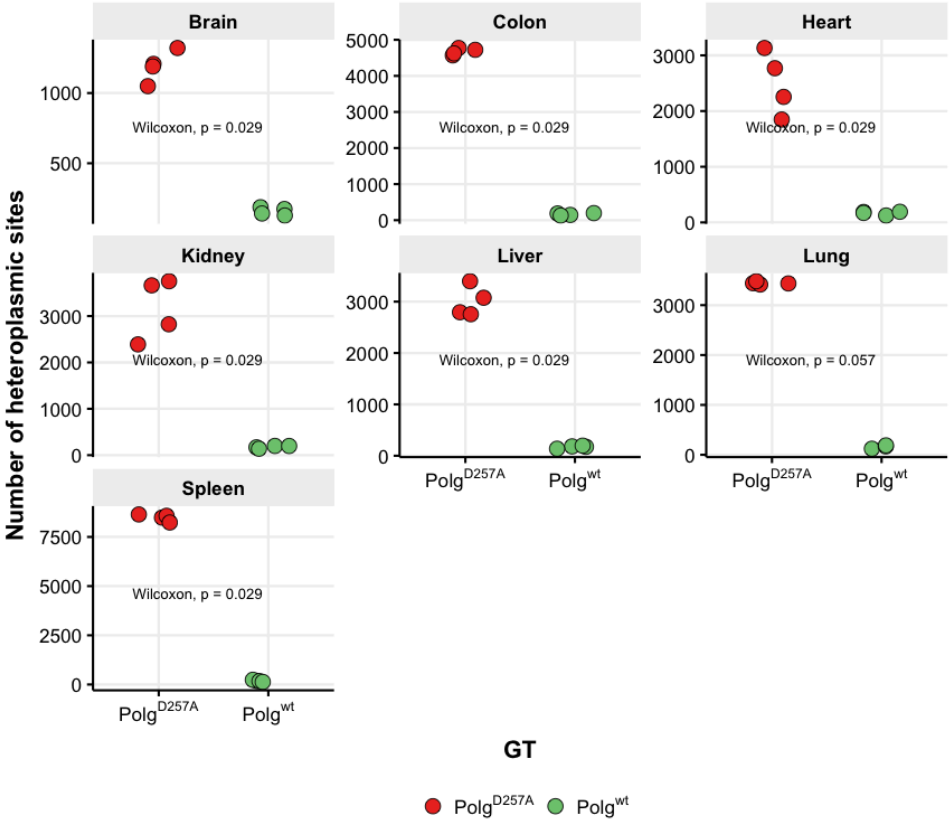

Supplementary figure 8. The number of heteroplasmic sites identified in each tissue of *Polg*$^{D257A/D257A}$ and *Polg*$^{wt/wt}$ mice (n=4). Wilcoxon rank-sum test.

S9

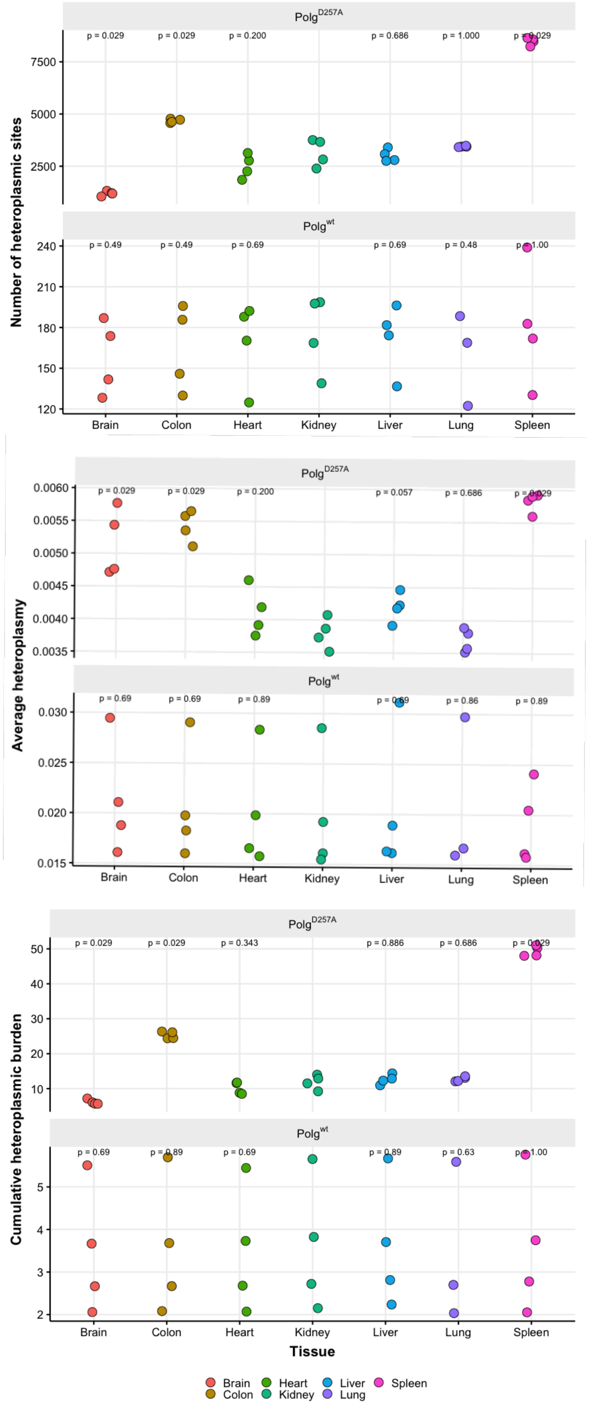

Supplementary figure 9. The tissue-specific pattern of mutation across *Polg*$^{D257A/D257A}$ and *Polg*$^{wt/wt}$ mice. a, Kidney, liver, colon, heart and lung had a similar number of heteroplasmic sites in *Polg*$^{D257A/D257A}$ mice with higher levels observed in spleen and lower levels found in brain. b, Colon and spleen had higher levels of average heteroplasmy than the other five tissues in *Polg*$^{D257A/D257A}$ mice. c, Cumulative heteroplasmic burden was higher in *Polg*$^{D257A/D257A}$ colon and spleen tissues and lower in brain than in heart, kidney, liver and lung. Wilcoxon rank-sum test with kidney acting as comparator for each statistical test.

S10

a

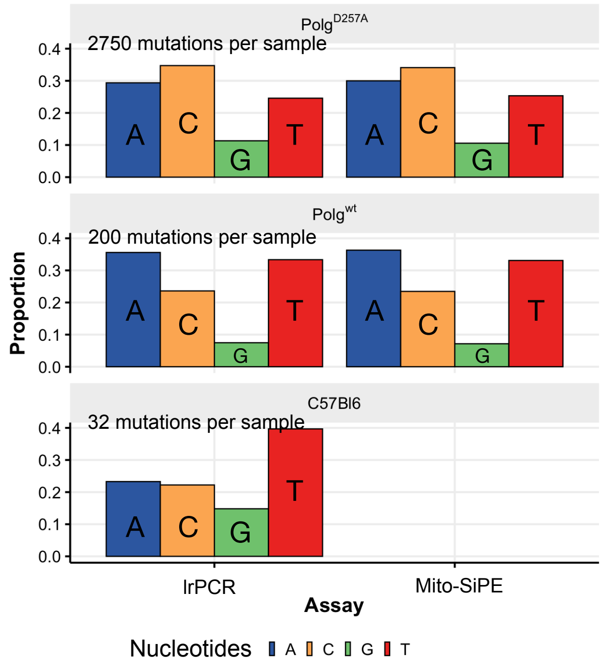

b

lrPCR

Alt

Ref

	A	C	G	T
A	0	0	40	21
C	0	0	5	86
G	24	0	0	0
T	7	5	0	0

Transitions	155
Transversions	33
Ts/Tv ratio	4.69697

Mito-SiPE

Alt

Ref

	A	C	G	T
A	0	2	31	23
C	0	0	4	88
G	23	0	0	0
T	11	11	0	0

Transitions	153
Transversions	40
Ts/Tv ratio	3.825

Supplementary figure 10. Characterisation of variants that were found across all samples using both lrPCR and Mito-SiPE. a, Long-range PCR amplification displayed similar results to Mito-SiPE in terms of proportion of mutations which occurred at each nucleotide in the mitochondrial genome. *Polg*$^{D257A/D257A}$ mice had a much larger number of mutations compared to *Polg*$^{wt/wt}$ and C57Bl6 wild-type mice. There was a significant difference in the mutations found in C57Bl6 between lrPCR and Mito-SiPE. b, Mutations that were present at a frequency >=10% heteroplasmy showed similar mutational profiles between lrPCR and Mito-SiPE. Although the Ts/Tv ratios appeared to be slightly different, this was not statistically significant (Chi-squared, p=0.43).

S11

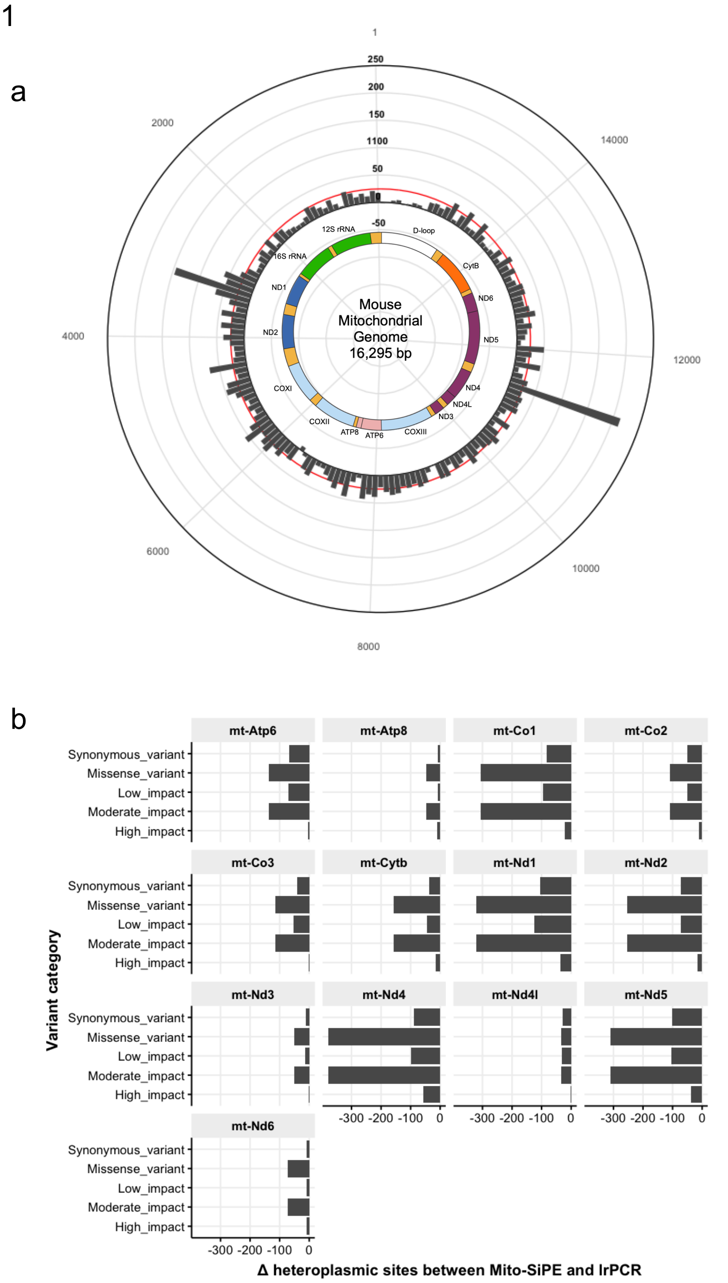

Supplementary figure 11. a, The difference in number of heteroplasmic sites identified using both methods plotted by location. The x axis represents the location in the mitochondrial genome (grouped as 100bp regions) and the y axis represents the difference in the number of heteroplasmic sites found in all samples using lrPCR and Mito-SiPE. Long range PCR amplification has more variants identified across the whole genome with a marked increase at locations where sequencing depth is reduced. The red line represents the average difference between the methods across the whole mitochondrial genome. b, Annotation of the variants using snpEff. Variants were annotated and characterised by their predicted effect. There was no obvious pattern between the difference of high, medium and low impact variants; however, more variants were identified using lrPCR in mt-Nd1 and mt-Nd4 genes. These genes are located in a region that has lower sequencing depth using lrPCR than Mito-SiPE.

S12

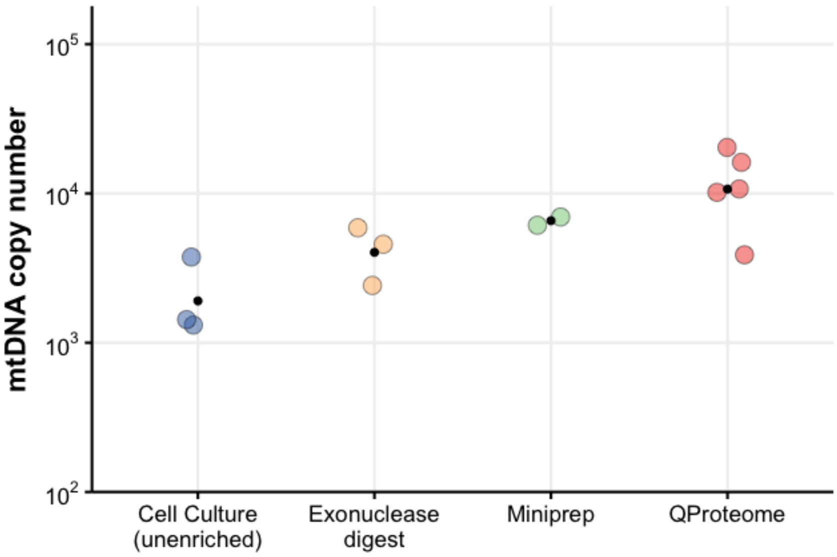

Supplementary figure 12. Relative mtDNA content (mtDNA copy number) of cell culture samples using sequence-independent methods. A qPCR assay that targeted the nuclear encoded β2-microglobulin and another assay that targeted the mitochondrial-encoded tRNA-leucine were used to calculate relative mtDNA content in cell culture samples that were enriched for mtDNA. These methods were compared to an unenriched cell culture sample. The level of enrichment was greatly reduced using these methods compared to Mito-SiPE, which was used on fresh mouse tissues. The y-axis shows the number of mitochondrial genomes present per haploid nuclear genome and the black point represents the mean. The x-axis displays the experimental groups (n=3 cell culture, n=3 exonuclease digest, n=2 miniprep and n=5 Qproteome).
