# Supplementary material for: Mito-SiPE is a sequence-independent and PCR-free mtDNA enrichment method for accurate ultra-deep mitochondrial sequencing
Source: Commun Biol. 2022 Nov 19;5:1269. doi: 10.1038/s42003-022-04182-2 (PMC9675811; doi:10.1038/s42003-022-04182-2)
Supplement: Supplementary file 3 — Description of Additional Supplementary Files [file 42003_2022_4182_MOESM3_ESM.pdf]

Description of Additional Supplementary Files

File name: Supplementary Data 1
Description: The following is the list of genes in the mitochondrial genome and the number of mutations that were identified in each using lrPCR and mtDNA preps. They are categorised by predicted effect.

File name: Supplementary Data 2
Description: The library complexity and fragment sizes of 15 randomly selected samples that were sequenced as calculated by Picard tools version 1.4.2.

File name: Supplementary Data 3
Description: The primers used for lrPCR of mouse mitochondrial DNA, qPCR assessment of mtDNA copy number from mouse DNA and qPCR assessment of mtDNA copy number from human DNA.

File name: Supplementary Data 4
Description: The source data used to create the graphs shown in this paper.
